# Tele-clinics in palliative care during the COVID-19 outbreak: Tertiary care cancer center experience

**DOI:** 10.15537/smj.2022.43.4.20210808

**Published:** 2022-04

**Authors:** Nabil A. Almouaalamy, Amal A. Jafari, Alaa M. Althubaiti

**Affiliations:** *From the Oncology Department (Almouaalamy), Princess Noorah Oncology Center, Ministry of National Guard - Health Affairs; from King Abdullah International Medical Research Centre (Almouaalamy, Althubaiti); from the College of Medicine (Almouaalamy, Althubaiti), King Saud bin Abdulaziz University for Health Sciences; and from the Nursing Department (Jafari), King Abdulaziz Medical City, Ministry of National Guard - Health Affairs, Jeddah, Kingdom of Saudi Arabia.*

**Keywords:** palliative care, telemedicine, COVID-19

## Abstract

**Objectives:**

To investigate the effect of tele-clinics on palliative care patients during the COVID-19 pandemic.

**Methods:**

This is a retrospective cross-sectional study (chart review) carried out from March 17, 2020, to September 16, 2020, included all patients who were booked into the palliative care clinic. Patients were assessed by the palliative nurse specialist for COVID-19 symptoms using the acute respiratory illness screening form and Edmonton Symptoms Assessment System, also identifies the needs of the patient. Data were analyzed to investigate the effect of tele-clinics on the patients regarding ER visits and admission.

**Results:**

A total of 167 individuals were analyzed and the results showed that 234 of 447 visits were virtual, supporting the increasing value of telemedicine. The number of virtual patients’ visits dropped slightly at the beginning of the pandemic (46.4% in March to 39.8% in July). Subsequently, it increased steadily to 72.2% in September. The choice of virtual/non-virtual visits for individuals with cancer diagnosis significantly depends on other factors. Code status, palliative patients or follow-up service, and the frequency of oncology center visits, admissions, or ER visits were crucial in explaining the means of receiving treatment.

**Conclusion:**

Virtual visits in palliative care are efficient means of decreasing the threat of COVID-19 contagion. It is recommended to increase the palliative care patients’ awareness of tele-clinics and their positive outcomes, particularly during the pandemic.

**T**he World Health Organization (WHO) defines palliative care as “an approach that improves the quality of life of patients and their families facing the problem associated with life-threatening illness, through the prevention and relief of suffering by means of early identification and impeccable assessment and treatment of pain and other problems, physical, psychosocial, and spiritual.”^1^

In the Kingdom of Saudi Arabia (KSA), palliative care service (PCS) is delivered to patients diagnosed with life-threatening illnesses; it aims to improve their quality of life by relieving suffering and managing pain and other symptoms.^2^ Palliative care service is a provision by an interdisciplinary team through different hospital stages, including inpatient-based, outpatient-based, and home health care within the healthcare system in KSA. Cancer is a major health problem in KSA and it is one of the leading causes of death, by 10%.^3^ The total number of newly diagnosed cancer cases reported to the Saudi Cancer Registry between January 1 and December 31, 2015, was 16,210.^4^

The use of telehealth for home care is on the rise and could provide a way to address the reports of challenges and meet the needs of patients who receive palliative care at home or in the hospital.^5^ Telehealth is defined as “the provision of healthcare remotely by means of a variety of telecommunication tools”.^5^ The delivery of telehealth can be interactive, in which patients and health care professionals exchange information or messages, or passive, in which neither recipient directly responds to the other.^6^

On December 31, 2019, the WHO regional office in China was informed on pneumonia cases of unknown origin in Wuhan City, Hubei Province.^7^ On January 7, 2020, the Chinese authorities announced they had identified a new virus associated with these cases.^5^ On March 2, 2020, the first COVID-19 case was recorded in KSA. Moreover, on March 11, 2020, the WHO categorized the COVID-19 outbreak as a pandemic.^8^ In the Ministry of National Guard - Health Affairs, all healthcare workers have been following the National Ministry of Health Readiness and National Guard Health Affairs Readiness strategies collaborating with the Infection and Prevention Control Department to manage suspected or confirmed cases.

Palliative care service continues to provide continuum service and maintains patient safety for all inpatient and outpatient settings, despite the COVID-19 pandemic. In a palliative outpatient setting, the tele-clinic project was initiated with the support of stakeholders in Ministry of National Guard-Health Affairs, Jeddah, Saudi Arabia to deliver a quality service that would not affect the patient’s safety or care while decreasing physical attendance in the outpatient area. Our study aims to investigate the impact of tele-clinics on palliative care patients during COVID-19 and the effect of that on emergency room (ER) visits and admissions as well.

## Methods

This study is a retrospective cross-sectional study (chart review) that investigated the impact of tele-clinics on palliative care patients during COVID-19 in Princess Noorah Oncology Center (PNOC), King Abdulaziz Medical City, Jeddah, Saudi Arabia from March 17, 2020 till end of September 30, 2020. The informed consent was waived due to the design of the study, and the study design was approved by the appropriate ethics review board. Ethical approval was obtained from the Institutional Review Board of the King Abdullah International Medical Research Center, National Guard Health Affairs, Riyadh, Saudi Arabia, with Memorandum Reference No. IRBC RJ20-140-J.

All patients aged 18 years or above who booked palliative care outpatient services from March 17, 2020, to September 16, 2020, were included in this retrospective review. The exclusion criteria were incomplete charts and privacy requests.

The researchers used the Edmonton Symptom Assessment System (ESAS) and acute respiratory illness screening form (ARI) during the study. Edmonton Symptom Assessment System is a tool designed to assess 9 common symptoms in patients with cancer (pain, tiredness, nausea, depression, anxiety, drowsiness, appetite, well-being, and shortness of breath). It has been psychometrically validated and translated into more than 20 languages.^9^ The ARI screening form is a locally developed screening tool used to triage patients, applying precautions, and testing for the symptoms for both MERS-CoV and COVID-19. It is composed of 2 categories: the first is the exposure risk, and the second is the clinical signs and symptoms. The sensitivity and specificity of the ARI are 56% and 79.4%.^10^

The COVID-19 case definition is a patient with acute respiratory illness (a sudden onset of at least one of the following: fever or recent history of fever, cough, and shortness of breath) or fever with gastrointestinal symptoms (nausea/vomiting and diarrhea).^11^ Individuals in physical contact (with or without symptoms) with a confirmed COVID-19 case in the last 14 days and any admitted patient with newly diagnosed pneumonia, either community-acquired pneumonia or hospital-acquired pneumonia, will include all cases of unexplained acute respiratory distress syndrome.^9^

### Palliative clinic

Princess Noorah Oncology Center, King Abdulaziz Medical City, Jeddah, Saudi Arabia is a tertiary cancer center with an 88-bed adult general oncology inpatient unit, a 22-bed bone marrow transplant unit, and a 32-bed pediatric hematology and oncology unit, including pediatric bone marrow transplant and specialized pediatric oncology emergency room. Palliative care service is an integrated service within the PNOC, where the inpatient beds are shared among all other services. Palliative care service has a hospital-wide consultation service, an outpatient clinic, and community care through the home health care program in King Abdulaziz Medical City, Jeddah, Saudi Arabia. Palliative services have 4 clinics per week: clinics for adult patients under PCS, new consultations, or follow-up patients referred from other specialties such as medical oncology, radiation oncology, hematology-oncology, gyne-oncology, internal medicine, general surgery, and gastroenterology. Patients visit palliative clinics for the management of symptoms such as pain and other physical symptoms and transfer of care; patients attending the clinic can be either palliative service patients or consultation/follow-up patients. Palliative clinics are managed by palliative consultants and patient care technicians in King Abdulaziz Medical City, Jeddah, Saudi Arabia. However, during the COVID-19 pandemic, palliative tele-clinic services were initiated, which aligned with the study period. All the patients in the study were booked for a physical visit, and was offered the virtual visit if they were stable and had controlled symptoms and accepted the concept of the virtual visit, or if the patient had an ARI score ≥6 the visit would be scheduled as a virtual only.

Palliative tele-clinics are managed by palliative nurse specialists and palliative consultants. Patients booked into the palliative clinics are contacted by a palliative nurse specialist one day before their appointment. The average call is 7 minutes per patient. These patients call on their registered numbers on the BESTCare system using an access code from PNOC. During the tele-clinic, patients are assessed for COVID-19 symptoms using the ARI screening form and ESAS, if ARI score is ≥6, the patient will be advised to communicate with local health authorities and infection control will be notified for further action and his visit would be scheduled as a virtual only. Subsequently, a palliative nurse specialist identifies the needs of the patient, such as symptom management, medication adjustments, refill medications, home supplies, or rescheduling the visit if patients are stable and have sufficient medication. Then, a palliative nurse specialist informs the palliative consultant regarding the patients’ needs. Patients who require symptom management and medication adjustment are contacted by a palliative consultant at their scheduled appointment on their contact number and provided with the treatment plan and prescription. For patients who need to refill medication, palliative consultants prescribe pills. They can come to the pharmacy to collect their medication if the prescription has an opioid. In the case of other drugs, the pharmacy makes arrangements with the primary care clinic that fits the individuals’ location, and they can collect them there. Otherwise, the medication can be sent to their home addresses via registered delivery companies, as per the patients’ preferences.

The source of data was the BESTCare Health Information System (HIS). The following data were retrieved from the palliative care outpatient BESTCare: age, gender, marital status, area of residence, code status, primary diagnosis, referral specialty, admission outcome, and COVID-19 case definition score.

The dependent variables are clinic attendance, admission, and ER visits during the study period. The independent variables are age, gender, area of residence, code status, diagnosis, the specialty of the patient most responsible physician, and COVID-19 case definition score.

### Statistical analysis

All patients who booked into the palliative care clinic during the study period were included. The data were described as median (range) for numerical variables and percentages for categorical variables. The Chi-square test or Fisher’s exact test was used to examine the associations between categorical variables, and the Mann-Whitney U test was used to compare non-parametric data. A stepwise forward multiple logistic regression was used to determine significant predictors of the type of visit. Odds ratios (ORs) with 95% confidence intervals were presented. Variables significantly associated with the type of visit in the bivariate analysis were entered into the multiple logistic regression analysis. Statistical significance was set at *p*<0.05, and data were analyzed using the SPSS database (IBMCorp, Armonk, NY, USA).

## Results

**Figure 1:**
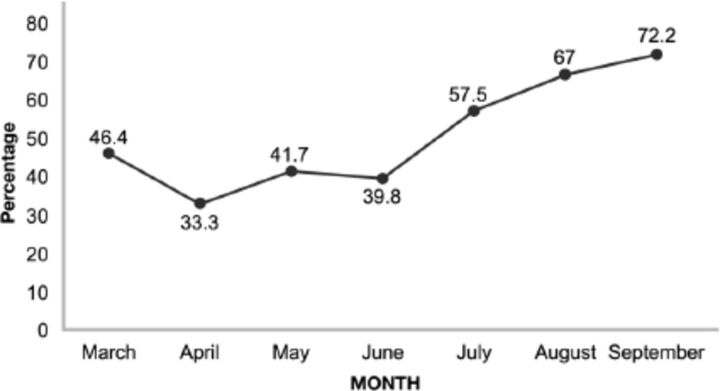
- Percent of virtual visits during COVID-19 period.

The total number of participants in the study was 167. The number of visits during the study period (March to September 2020) was 447. The median number of visits per patient was 2 (range, 1-10). Of 167 patients, 62 (37.1%) had only one visit during the study period, and 28 (16.8%) had 2 visits during the study period. The median age was 68 (18-108) years, 52.7% were male, 79% were married. The most common code statuses were full code (n=80, 47.9%), followed by support care (n = 53, 31.7%). The baseline characteristics of patients are provided in Table 1. The median ARI score was 0 (range, 0-12). Most patients had an ARI score of zero (74.3%). More than half of the visits were virtual visit (n=234, 52.3%) (Table 2). There was an increasing trend in the percentage of virtual visits during the COVID-19 period (Figure 1).

**Table 1:** - Demographics and clinical characteristics of the study participants (N=167).

Characteristic	Descriptive statistics
Age, years; median (range)	68 (18-108)
***Gender***	
Female	79 (47.3)
Male	88 (52.7)
***Marital status***	
Married	132 (79.0)
Other	35 (21.0)
***Area of residency***	
Western region	152 (91.0)
Other	15 (9.0)
***Code status***	
Full code	80 (47.9)
Support care	53 (31.7)
Comfort care	34 (20.4)
***Diagnosis***	
Cancer	165 (98.8)
Non-cancer	2 (1.2)
***Palliative / follow-up patients***	
Palliative patient	46 (27.5)
Follow-up patient	121 (72.5)
Number of visits; median (range)	2 (1-10)
Number of admissions; median (range)	1 (0-9)
No admission	65 (38.9)
One admission or more	102 (61.1)
Number of ER visits, median (range)	1 (0-21)
No ER visit	62 (37.1)
One or more ER visit	105 (62.9)
***Specialty***	
Medical oncology service	72 (43.1)
Gynae-oncology	17 (10.2)
Hematology	19 (11.4)
Radiation	28 (16.8)
Non-oncology service	31 (18.6)

Values are presented as number and percentages (%).

**Table 2:** - Acute respiratory illness score and type of visits recorded during seven months of COVID-19 period in the palliative center.

Characteristics	Descriptive statistics
ARI score, median (range)	0 (0–12)
ARI score	
0	332 (74.3)
1-3	92 (20.6)
>3	23 (5.1)
***Type of visit***	
Virtual visit	234 (52.3)
Non-virtual visit	213 (47.7)
Show up	90 (42.2)
Walk-in, inpatient, or other	123 (57.8)

Total is computed over the study period (N=447). Values are presented as number and percentage (%) unless otherwise stated. ARI: acute respiratory illness

### Virtual versus non-virtual visits

To compare the characteristics of patients between the types of visits, virtual or non-virtual, we divided the patients into 2 groups: those who had at least one virtual visit and those who never had any virtual visit. Table 3 presents the characteristics of the patients according to the type of visit.

**Table 3:** - Comparison of patients’ characteristics and clinical variables between types of visit groups.

Characteristics	Type of visit		*P*-value
	At least one virtual visit (N=124)	No virtual visit (N=43)	
Age, years; median (range)	68 (18-108)	66 (36-88)	0.74*
***Gender***			
Female	55 (69.6)	24 (30.4)	0.20^†^
Male	69 (78.4)	19 (21.6)	
***Marital status***			
Married	99 (75)	33 (25)	0.67^†^
Other	25 (71.4)	10 (28.6)	
***Area of residency***			
Western region	112 (73.7)	40 (26.3)	0.76^†^
Other	12 (80)	3 (20)	
***Code status***			
Full code	67 (83.8)	13 (16.3)	<0.001^†^
Support care	41 (77.4)	12 (22.6)	
Comfort care	16 (47.1)	18 (52.9)	
***Diagnosis***			
Cancer	123 (74.5)	42 (25.5)	0.45^‡^
Non-cancer	1 (50)	1 (50)	
***Palliative / follow-up patients***			
Palliative Patient	29 (63)	17 (37)	0.041^†^
Follow-up Patient	95 (78.5)	26 (21.5)	
Number of visits, median (range)	3 (1-10)	1 (1-6)	<0.00*
***Number of admissions***			
No admission	56 (86.2)	9 (13.8)	0.005^†^
One admission or more	68 (66.7)	34 (33.3)	
***Number of ER visits***			
No ER visit	52 (83.9)	10 (16.1)	0.029^†^
One or more ER visit	72 (68.6)	33 (31.4)	
***Specialty***			
Medical oncology service	55 (76.4)	17 (23.6)	0.17^‡^
Gyne-oncology	11 (64.7)	6 (35.3)	
Hematology	17 (89.5)	2 (10.5)	
Radiation	22 (78.6)	6 (21.4)	
Non-oncology service	19 (61.3)	12 (38.7)	

Values are presented as number and percentage (%) unless otherwise stated. *Mann-Whitney U test for independent samples. ^†^Pearson’s Chi-square test, ^‡^Fisher’s exact test

The results showed that code status, palliative or follow-up status, attendance, admissions, and ER visits, were significantly associated with the type of visit. There is a decreasing trend towards virtual visits from full code (83.8%) to comfort code (47.1%). Palliative service patients were more likely to have no virtual visits (37% versus [vs.] 21.5%). The median number of visits was higher for those who had at least one virtual visit compared to those who had no virtual visits (*p*<0.001). Furthermore, patients with one or more admissions (33.3% vs. 13.8%, *p*=0.005) and one or more ER visits (31.4% vs. 16.1%, *p*=0.029) more likely had not experienced any virtual visits. Age, gender, marital status, area of residence, diagnosis, ESAS score and specialty were not significantly associated with the type of visit.

### Multiple logistic regression

Code status and the number of visits were found to be significantly and independently associated with the type of visit. Those in comfort care were approximately 6 times more likely (95% CI 2.33-14.91) to have no virtual visits compared to those in full code status. Moreover, patients with a decreased number of visits were approximately half as likely to have a virtual visit compared to patients with a larger number of visits (Table 4).

**Table 4:** - Multiple logistic regression analysis for variables associated with the type of visit.

Characteristics	OR	95% CI	*P*-value
***Code status***			
Support care	1.3	0.5–3.2	0.60
Comfort care	6.0	2.3–14.9	<0.001
Number of visits	0.6	0.5-0.9	0.001

OR: odd ratio, CI: confidence interval

### Comparison of number of admissions and number of ER visits by code status

Patients with full code status were relatively less likely to be admitted (69.2 % vs. 34.3%, *p*<0.001) or go to the ER (61.3% vs. 40%, *p*=0.022) (Table 5).

**Table 5:** - Comparison of number of admissions and number of ER visits by code status over the study period.

Characteristics	Code status			*P*-value
	Full code	Support code	Comfort code	
***Admission***				
None	45 (69.2)	12 (18.5)	8 (12.3)	<0.001
One or more admissions	35 (34.3)	41 (40.2)	26 (25.5)	
***ER visits***				
None	38 (61.3)	13 (21)	11 (17.7)	0.022*
One or more	42 (40)	40 (38.1)	23 (21.9)	

Values are presented as number and percentage (%). Admission and emergency room (ER) visits over the study period. Only significant comparisons are presented. *Pearson’s Chi-square test

## Discussion

This study aimed to investigate the effect of tele-clinics on palliative care patients during the COVID-19 pandemic in PNOC. To serve the primary goal, we analyzed clinic attendance, hospital admissions, and ER visits of 167 participants during the study period. Overall, the findings support previous research on the value of telemedicine in palliative care.

First, the data analysis revealed that all patients diagnosed with cancer visited PCS at least once from March to September 2020 for one medical specialty. This agrees with the study by Al-Shahri et al^2^ that affirms frequent visits by cancer patients to palliative care centers. Al-Shahri et al^2^ argue that ineffective management of palliative care reduces its accessibility and affordability, increasing the suffering of the patients and their families. Therefore, virtual interactions should become an integral part of the new models of care in Saudi Arabia.^12^ Our findings reveal the relevance of palliative care for individuals diagnosed with cancer and telemedicine as a beneficial way to increase people’s access to the required services.

Although patients continued attending the oncology center in person, the number of virtual visits was higher than that of non-virtual visits. The results show that 234 of 447 visits were virtual, supporting the increasing value of telemedicine. Interestingly, the number of virtual patients’ visits dropped slightly at the beginning of the pandemic (46.4% in March to 39.8% in July). Subsequently, it increased steadily to 72.2% in September. Additionally, there was a marked decrease in non-virtual visits (47.7%) throughout the study period that is similar to what has been identified by Reddy et al.^13^ The rapid spread of COVID-19, which requires strict social distancing, limited contact, and so on, can explain this tendency. Grewal et al^14^ add that healthcare and palliative care systems prioritize advanced care planning during the COVID-19 outbreak, ensuring patient safety, continuing the proper treatment, and minimizing contagion. Few studies have researched the association between COVID-19 and telemedicine; therefore, future research should focus on this topic. More visits to tele-clinics than face-to-face interactions had positive outcomes on patients’ affordability of treatment and quality of life. However, similar studies showed that care providers and patients are forced to use telephone visits because of the inability of patients to connect to the audio-video visit, primarily because of user errors, which prompted a telephone visit.^15^ Additionally, Lally et al^16^ have found that a virtual visit is not an option for many patients because they lack the technical capability, and those patients were often non-native English speakers, socioeconomically disadvantaged, or older. The data are supported by AlAzab and Khader^17^ or Inglis et al,^18^ who emphasize that virtual visits decrease expenditures for PCSs and improve the quality of life. Our findings also agrees with the research by AlShehery et al^19^ that virtual clinics ensure patient safety, reduce the risk of contagion, increase the individuals’ satisfaction with services, and improve communication between the staff and patients’ families.

Finally, the findings show that the choice of virtual/non-virtual visits for individuals with cancer diagnosis significantly depends on other factors. Code status, palliative or follow-up service, and the frequency of oncology center visits, admissions, or ER visits were crucial in explaining the means of receiving treatment. Thus, patients with a full code status were more likely to use tele-clinic services than comfort care individuals were. Consenting to all possible interventions to prolong their lives, they may regard virtual consultations as another beneficial way to receive timely treatment/guidance. Moreover, a relatively higher number of virtual visits implies that tele-clinics facilitate the accessibility of outpatient palliative care. These results are consistent with the study by Steindal et al,^20^ confirming palliative care patients have relatively higher access to treatment from healthcare professionals. Such patients feel more comfortable managing their treatment or receiving counseling in the home environment because it enhances their safety and quality of life. A similar finding by Berlin et al^21^ show that patients experiencing virtual visits are more likely to consider them than in-person visits and request them for their future appointments. Consequently, telemedicine seems to be a beneficial solution for cancer patients with low access to PCSs in Saudi Arabia, as described by Al-Shahri et al.^2^

Although palliative care patients chose tele-clinics (23.4%) at least once, they were still more likely to receive inpatient treatment (39.5%). Hospital admission and ER visits also reduce the opportunity to receive guidance/support via technology. This implies that the severity of the symptoms requires inpatient treatment in the oncology center setting. If an individual’s physical condition allows them to stay at home instead of a hospital, tele-clinics become a relevant option. A similar tendency can be observed in a study by Clark et al,^22^ where remote monitoring programs do not reduce admissions to hospitals for diverse causes. In a systematic review study, Hancock et al^23^ find only an insignificant effect of virtual clinics in reducing admissions to clinics and emergency care. Nevertheless, Clark et al^22^ and Inglis et al^18^ claim that telemedicine has decreased admissions of patients with chronic heart failure and mortality. Thus, future studies should research when tele-clinics may reduce hospital admissions and diseases when they are less effective. A large randomized clinical trial is recommended to generalize the findings and explain the inconsistent results concerning the impact of virtual visits on all-cause admissions.

### Study limitations

This is it is a retrospective study with a small sample size, although we included all the patients from our clinic besides additional walk-in patients. Future studies should employ larger sample sizes and a longitudinal design to increase the ecological validity of the study findings. Additionally, as the study was based on experiences from one center, its generalizability to other settings could be limited.

In conclusion, palliative tele-clinic and virtual visits are efficient means to decrease the threat of COVID-19 contagion. Moreover, tele-clinics will improve communication between the staff and patients’ families, providing timely treatment, guidance, and counseling, providing timely treatment, guidance, and counseling. Undoubtedly, the severity of patients’ physical conditions, with multiple admissions and ER visits, along with being a palliative patient, leads to refusal of virtual visits. Therefore, it is recommended to increase the palliative care patients’ awareness of tele-clinics and their positive outcomes, particularly during the pandemic.
